# Glutamine Peptides: Preparation, Analysis, Applications, and Their Role in Intestinal Barrier Protection

**DOI:** 10.3390/nu17061017

**Published:** 2025-03-14

**Authors:** Jinyu Wang, Yating He, Zedan Liu, Xiaolan Liu, Yan Jing

**Affiliations:** Key Laboratory of Corn Deep Processing Theory and Technology of Heilongjiang Province, College of Food and Bioengineering, Qiqihar University, Qiqihar 161006, China

**Keywords:** glutamine peptides, intestinal barrier function, intestinal health

## Abstract

**Background:** Glutamine peptides refer to a series of peptides containing glutamine, and the activity of glutamine peptides is characterized by the content of non-nitrogen terminal glutamine in the peptide. It has been found that glutamine peptides are a stable substitute for glutamine monomer, and they are increasingly studied in nutrition and physiology due to their functional properties. **Methods:** An extensive search of the literature was conducted in the PubMed, Web of Science, Scopus, and Google Scholar databases up to December 2024. Inclusion criteria focused on the role of glutamine peptides in intestinal health, and the included literature was screened and summarized. **Results**: This study systematically reviews the current status of research on the preparation, analysis, applications of glutamine peptides and their role in intestinal barrier protection. Furthermore, the challenges faced by the current research and the development direction in the future are discussed. **Conclusions**: Glutamine peptides can play a role in protecting the intestinal barrier by regulating tight junctions, mucin, inflammatory response, and intestinal flora. In addition, further and intensive investigations are urgently required to address the current challenges pertaining to the structure–activity relationships of glutamine peptides and their transport and absorption mechanism in the gut. This review contributes to a better understanding of the mechanism of glutamine peptides to protect intestinal barrier function and also provides a reference for the development of functional foods with protective effects of intestinal barrier function.

## 1. Introduction

The intestinal tract is an important digestive and immune organ of the human body. The absorption of most nutrients and the excretion of food toxins depend on the intestinal tract, and a large number of immune cells are also distributed in the intestinal tract [1]. The intestinal barrier is an important basis for intestinal function, which can strictly control the exchange of substances between the host and the external environment [2,3]. Compromised barrier integrity not only impairs nutrient digestion and absorption but also weakens the body’s disease resistance, increasing susceptibility to pathogenic microorganisms and triggering various systemic diseases [4,5]. Therefore, protecting intestinal barrier function is essential for safeguarding intestinal health and reducing the incidence of intestinal and related systemic disorders.

Clinically, enteral nutrition is the primary approach for protecting intestinal barrier function [6,7,8]. Currently, commonly used enteral nutrients include glutamine, growth hormone, and fatty acids [9,10]. Recent studies highlight the unique and irreplaceable role of glutamine in preserving intestinal structural and functional integrity, particularly under both physiological and pathological conditions [11]. Glutamine exerts critical protective effects on intestinal barrier function, surpassing those of other amino acids, by serving as a primary energy source for enterocytes and modulating key cellular repair pathways [12].

**Figure 1:**
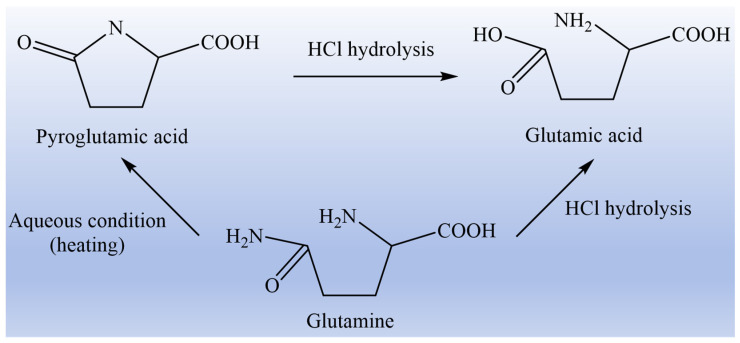
Schematic diagram of the conversion of glutamine to glutamic acid and pyroglutamic acid.

Based on the various physiological functions of glutamine, corresponding food, drugs, or functional health products can be researched and developed according to the different needs of different people [13,14]. Despite its widespread pharmaceutical use, glutamine remains underutilized in food products due to the limitations of glutamine monomer itself, which is reflected in the following three aspects: first of all, glutamine monomer is easily converted to toxic pyroglutamic acid under heating conditions, and it is also extremely unstable in aqueous solution, which is easily decomposed to glutamic acid and ammonia. The transformation diagram of glutamine to glutamic acid and pyroglutamic acid is shown in Figure 1. Secondly, glutamine has low solubility in aqueous solution due to its side chain having no charge. Third, the human body is prone to allergic reactions to glutamine, such as nausea, vomiting, shock, and poor adaptability [15,16,17]. Therefore, it is of critical practical significance to find suitable and stable substitutes for glutamine.

Studies have found that some food-derived bioactive peptides, especially glutamine peptides, have shown promise in protecting intestinal barrier function and promoting gut health. Their advantages, including excellent water solubility, high stability, enhanced bioavailability, and low allergenicity, make them a stable alternative to glutamine monomers [18,19,20]. Although in recent years glutamine peptides have been increasingly studied in nutrition and physiology, the systematic summary of their preparation, analysis, application, and protective effect on intestinal barrier function is limited. Therefore, in this paper, the preparation method of glutamine peptides, the determination method of glutamine in peptides, the application of glutamine peptides, and the protective effect on intestinal barrier function are systematically reviewed. At the same time, the problems and challenges faced by current research are discussed, and the future research direction is prospected. In conclusion, the purpose of this review is to understand the role and significance of glutamine peptides in human health and to provide directions for future research work.

## 2. Methodology

In order to perform a comprehensive literature review on the role of glutamine peptides in the intestine, an extensive search of the literature was conducted through the PubMed, Web of Science, Scopus, and Google Scholar databases up to November 2024. The keywords included “glutamine”, “glutamine peptides”, “intestinal barrier”, “intestinal health”, “mechanical barrier”, “chemical barrier”, “immunological barrier”, “biological barrier”, “intestinal inflammation”, and “intestinal flora”. Articles not published in English or lacking full text were excluded.

## 3. Overview of Glutamine Peptides

### 3.1. Preparation Method of Glutamine Peptides

Bioactive peptide is a fragment of protein with biological activity, which is usually buried in the complex structure of parent protein without physiological activity [21,22]. After processing or digestion, a variety of peptides with physiological activities can be released from maternal protein, affecting human health. The structural complexity of proteins allows for virtually infinite possibilities in bioactive peptide sequences [23]. Furthermore, their biological activity depends on physicochemical properties, such as molecular weight, amino acid composition, sequence, and spatial conformation [24,25]. Consequently, the preparation methods of bioactive peptides play a decisive role in determining their functional activity.

#### 3.1.1. Chemical Synthesis

**Figure 2:**
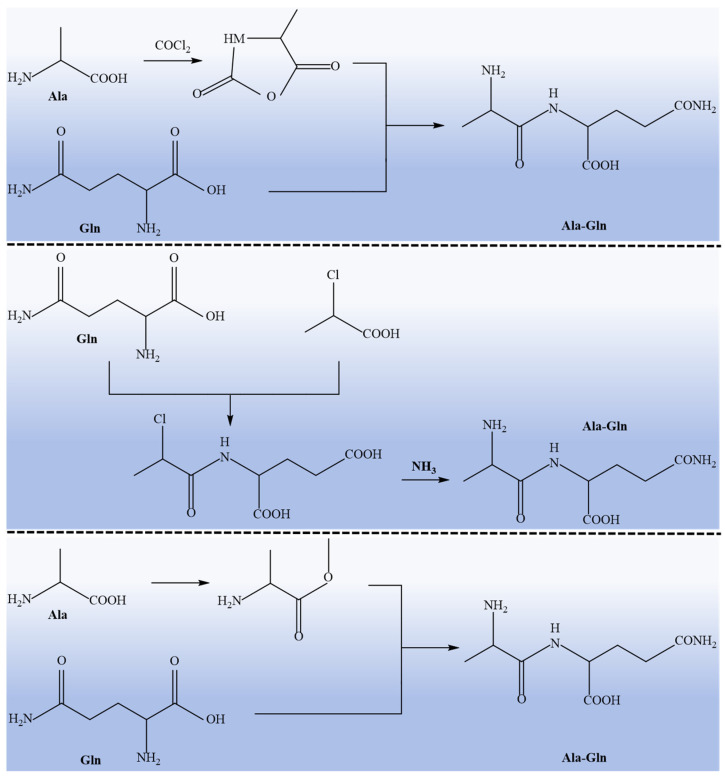
Chemical synthesis process of alanyl-glutamine (Ala-Gln) according to Yagasakia et al [29].

There are two main methods for the preparation of glutamine peptides: one is chemical synthesis, which combines glutamine with other amino acids to synthesize glutamine peptides according to needs. The commonly used synthesis methods are liquid-phase synthesis and solid-phase synthesis. The liquid-phase synthesis method depends on the liquid environment and is mostly used for short peptide synthesis [26]. Compared with liquid-phase synthesis, solid-phase synthesis is the preferred method for bioactive peptide synthesis because of its simple process and high synthesis efficiency [27]. At present, two dipeptides, alanyl-glutamine (Ala-Gln) and glycine-glutamine (Gly-Gln), are mainly used in domestic and foreign markets (Figure 2) [28,29]. However, the chemical synthesis of glutamine peptides faces challenges, such as high costs, complex processes, and safety concerns, due to toxic byproducts and side effects [30]. Therefore, the development of a low-cost, easy-to-operate, and environmentally friendly synthesis technology is of critical importance for the targeted synthesis and application of glutamine peptides.

#### 3.1.2. Enzymatic Hydrolysis

**Figure 3:**
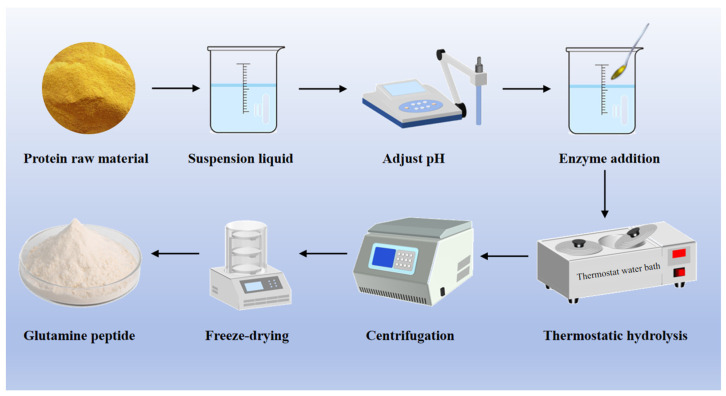
Preparation process diagram of glutamine peptide by enzymatic hydrolysis.

The second method for preparing glutamine peptides is the enzymatic hydrolysis of plant protein, mainly through the enzymatic hydrolysis of proteins to obtain polypeptides containing glutamine, and common raw materials for preparation are wheat, corn, potato, and so on (Figure 3) [31]. The yield and purity of glutamine peptides prepared by enzymatic hydrolysis are related to the preparation process, mainly influenced by the type of enzyme and hydrolysis conditions [32]. In 1992, Soichi Tanabe, a Japanese scholar, first used Molsin (XIII) type protease and Actinase E protease to hydrolyze wheat gluten protein to produce glutamine peptide, and the final non-nitrogen terminal Glx (glutamine and glutamic acid) content of the product reached 50.33 g/100 g [33]. In 1999, a nutrient solution rich in glutamine active peptides was developed [34]. As people paid more and more attention to food and drug safety, researchers gradually began to pay attention to the preparation of glutamine peptides by enzymatic hydrolysis of plant protein and conducted a series of studies. Food-grade proteases commonly used for the preparation of bioactive peptides (Table 1) mainly include animal proteases, plant proteases, and microbial proteases [35,36]. Alcalase, an endopeptidase with broad specificity for peptide bonds, exhibits minimal hydrolytic activity toward the amide groups in glutamine. Compared to other proteases, it demonstrates distinct advantages in the production of glutamine peptides [37]. In addition, different proteases have great differences in performance, enzymatic hydrolysis efficiency, action site, and enzymatic hydrolysis conditions due to their different sources [38]. Therefore, glutamine peptides can be prepared by enzymatic method with appropriate protease according to the characteristics of raw materials and actual conditions. However, in the process of enzymatic hydrolysis, not only glutamine-rich peptides can be obtained but also potentially allergenic peptides and hydrophobic peptides can be released, resulting in a bitter taste of the hydrolysate. Therefore, it is necessary to balance the relationship between hydrolysis degree and sensitization of bioactive peptides when preparing glutamine peptides by enzymatic hydrolysis.

Chemical synthesis and enzymatic hydrolysis represent two effective methods for glutamine peptides production; their respective advantages and limitations are comparatively outlined in Table 2. Owing to the operational complexity and elevated costs inherent to chemical synthesis, enzymatic hydrolysis is widely recognized as the most efficacious approach for industrial-scale manufacturing of glutamine peptides.

### 3.2. Method for Determination of Glutamine in Glutamine Peptides

It is well known that glutamine will undergo a conversion reaction during the routine detection of amino acids, so it cannot be effectively determined by the conventional amino acid analysis method (Table 3). At present, there are three main methods for the determination of glutamine content in glutamine peptides. The first method is enzymatic hydrolysis, which first hydrolyzes glutamine into glutamic acid and ammonia and then indirectly determines the content of glutamine by detecting the amount of ammonia [39]. There are two kinds of enzymes used, namely glutaminase and glutamine synthetase, in which glutaminase specifically acts on the carboxyl-terminal glutamine of peptide, while glutamine synthetase specifically acts on the amino-terminal or internal glutamine residue. Therefore, the two enzymes should be used at the same time in the determination. In addition, there are some limitations in the assay process, in which glutaminase can act on pentapeptide or even more, while glutamine synthetase can only act on tetrapeptide at most. Therefore, the enzyme hydrolysis method is often used for the determination of short peptides [40]. The second method is gene or sequence analysis based on cDNA technology and Edman sequencer. However, this method is only suitable for the analysis of glutamine content in pure protein or peptide samples, and the content of glutamine in the mixture can not be determined. Moreover, the detection cost of this method is relatively high, and the operation is complicated [41]. The third method is the BTI derivative method established by Kuhn in 1996 [42]. Its principle is that the non-*N*-terminal glutamine reacts with bis (1, 1-trifluoroacetic acid) iodobenzene (BTI) to produce L-2, 4-diaminobutyric acid (DABA), which will not be hydrolyzed into glutamic acid during acid hydrolysis, and the glutamine that does not react with BTI will be hydrolyzed into glutamic acid when it is hydrolyzed. Therefore, the content of glutamine can be calculated indirectly by comparing the content of glutamic acid in the two hydrolyzed products, or the content of glutamine can be calculated by detecting the content of DABA by HPLC. In addition, the BTI derivative method can only protect non-*N*-terminal glutamine, so the glutamine determined by this method is called effective glutamine. This method is also a common method for the determination of glutamine content in peptides.

### 3.3. Application of Glutamine Peptides

Based on the various physiological functions and nutritional properties of glutamine peptides, as well as their strong stability, solubility, and good absorbability, glutamine peptides can be applied in food, medicine, feed, and other fields. Scientists have combined glutamine with other drugs to make a new anti-ulcer drug, marzulene, which has been circulated in China. Clinical studies have found that L-glutamine sodium citrullinated granules, namely marzulene, have a significant therapeutic effect on hemorrhagic colitis [43]. The patient with eosinophilic gastritis ulcer improved significantly after receiving symptomatic treatment with L-glutamine sodium citrullinated granules [44]. At present, glutamine peptide has not been used as a drug for treating gastrointestinal diseases alone. Glutamine peptides prepared by enzymatic hydrolysis is naturally non-toxic and will not cause adverse reactions to the human body [45,46]. If it is developed into a product for preventing or treating gastrointestinal diseases, it will have a broad market prospect. In addition, some plant protein raw materials are fermented by special strains to produce small peptides rich in glutamine, which can be used as feed directly or as feed additives, and can effectively improve the weaning stress or oxidative stress response of animals [47,48]. Li et al. found that dietary alanyl-glutamine dipeptide can significantly improve the growth performance of juvenile yellow catfish and has positive promoting effects on the improvement of antioxidant capacity, immune response, and stress resistance of juvenile yellow catfish [49]. In the food industry, glutamine peptides can be added to drinks to develop sports drinks with the function of promoting exercise recovery, increasing immunity, and protecting the gastrointestinal barrier. Sawaki et al. found that glutamine peptide has an obvious anti-fatigue effect. After oral administration of glutamine peptide, plasma glutamine and branched-chain amino acid (BCAA) content increased significantly, and Trp/BCAA ratio decreased significantly [50]. With growing public attention to food nutrition and safety, the development of glutamine peptide products possessing multiple physiological functions is expected to demonstrate promising growth prospects in industries including food, pharmaceuticals, and animal feed.

## 4. Protective Effect of Glutamine Peptides on Intestinal Barrier Function

**Figure 4:**
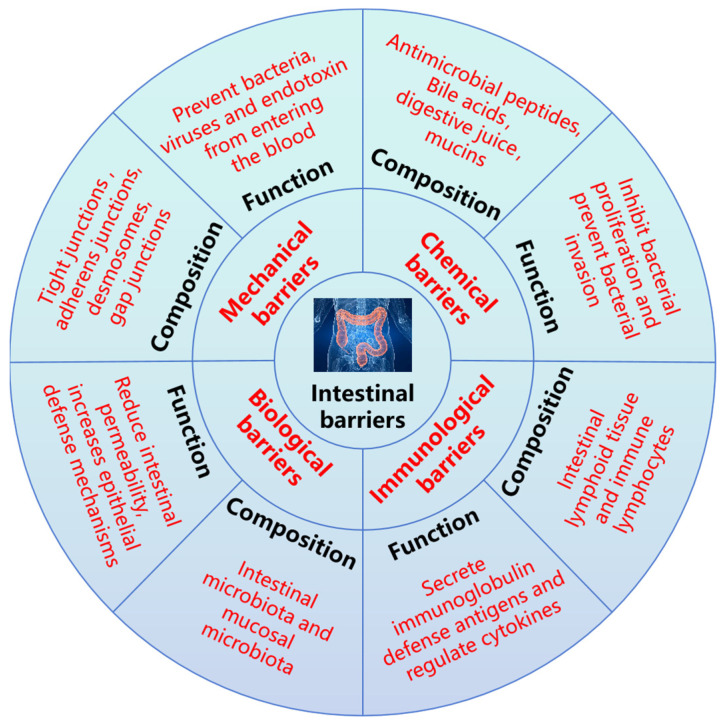
Composition and function of intestinal barrier.

The intestinal barrier can be generally divided into the mechanical barrier, chemical barrier, immunological barrier, and biological barrier (Figure 4). These barriers have corresponding components, which play a vital role in maintaining intestinal environmental homeostasis and hindering exogenous pathogens, and they are also congenital barriers to protect body health [51,52,53]. Under normal circumstances, these barriers cooperate to form a relatively complete defense system so that the internal environment of the body remains relatively stable, thus maintaining the normal life activities of the body.

The integrity of intestinal barrier function relies on the continuous mucosal layer and a well-functioning intestinal mucosal support system. When subjected to external stressors, such as chemotherapy, prolonged parenteral nutrition, starvation, trauma, or surgery, the mucosal integrity and support systems are compromised, leading to mucosal atrophy, dysbiosis of gut microbiota, and increased intestinal permeability, thereby resulting in intestinal barrier dysfunction [54]. Under normal physiological conditions, damage or abnormality of any of the four barriers that constitute the intestinal barrier may lead to intestinal barrier dysfunction. Abnormal intestinal barrier function will not only cause the emergence of new diseases but also aggravate the severity of existing diseases. For instance, intestinal barrier dysfunction can easily lead to intestinal bacterial translocation or enterotoxemia, thus inducing or aggravating systemic inflammatory response syndrome (SIRS) or multiple organ dysfunction syndrome (MODS), even causing multiple organ failure (MOF), and forming a vicious cycle [55]. Therefore, the protection of intestinal barrier function and timely intervention of abnormal intestinal barrier function are of great significance to block the basic vicious cycle and promote the recovery of diseases. As shown in Table 4, a series of glutamine peptides or protein hydrolysate with glutamine-rich peptides play an important role in intestinal barrier protection.

### 4.1. Protective Effect of Glutamine Peptides on Mechanical Barrier

The mechanical barrier, also known as physical barrier, constitutes the most critical component of the intestinal barrier. Its physiological structural basis consists of the mucosal epithelium, lamina propria, and muscularis mucosae. The connections between cells are primarily composed of tight junctions, adhesion junctions, and desmosomes. Under normal conditions, intestinal epithelial cells are tightly arranged through cell junctions, which can effectively block bacteria, viruses, and endotoxins from entering the blood. Tight junctions are composed of a variety of transmembrane and cytoplasmic-related proteins, including zonula occludens (ZOs), transmembrane proteins (Claudin), Occludin, and junction adhesion molecules (Jams) [67]. These proteins interact with each other and with the cytoskeleton to form complex structures that serve as barriers. The biological functions of tight junctions are mainly in two aspects: one is to maintain the permeability barrier function, which can not only regulate the transport of small molecules, ions, and other cross-cellular paracellular pathways but also prevent the entry of large molecules into cells. The second is to maintain cell polarity function, by restricting the free flow of proteins and lipids in different liquid spaces of cells, to maintain the balance of cell apical and basal spaces.

Studies have shown that bioactive peptides, especially glutamine peptides, have the ability to regulate tight junctions and improve physical barriers. For example, the dipeptide alanyl-glutamine protected intestinal barrier function against acute exhaustive exercise in rats by reducing intestinal permeability and regulating claudin-2 mRNA expression [68]. Similar results have been observed during in vivo experiments of piglets. Dietary Ala-Gln supplementation could up-regulate the mRNA expressions of Claudin-1, Occludin, ZO-1 and the protein levels of Occludin, ZO-1 in jejunal mucosa, indicating Ala-Gln supplementation could promote the functions of intestinal mucosa barriers in piglets [69]. In a Caco-2 cell model, collagen peptide (GPSGPQGSR) showed more potent activity in reducing TNF-α-induced barrier dysfunction compared to Gln [56]. Glutamine peptide (NPWDQ), a casein-derived short peptide, can enhance intestinal barrier function by up-regulating the expression of Occludin [70,71]. Tight junctions are generally regulated by a variety of proteins, such as Myosin light chain kinase (MLCK), phospholipase C, protein kinase A, and mitogen-activated protein kinases, among which MLCK plays a key role in the regulation of tight junctions [72]. MLCK has been regarded as a central regulator of intestinal barrier function in response to a wide range of stimuli [73]. It has been reported that inhibition of the NF-κB-mediated MLCK-MLC signaling pathway can improve intestinal barrier dysfunction in mice [74]. Liu et al. found that supplementation of Ala-Gln in the basic diet significantly up-regulated the expression of intestinal tight junction proteins claudin-1, occludin, and ZO-1, down-regulated the expression of NF-κB and MLCK, and inhibited the NF-κB-MLCK signaling pathway in turbot [75]. However, Li et al. studied the transport and absorption of shrimp polypeptide, and found that QMDDQ achieved complete absorption by mediating the MLCK signaling pathway, reversible opening of ZO-1 and Occludin. Namely, QMDQQ activated the MLCK signaling pathway and decreased the expression of tight junction proteins ZO-1 and Occludin. This seems to be the opposite of what was described above. Therefore, the specific regulatory mechanism of glutamine peptide promoting tight junction protein expression remains to be further explored [76].

### 4.2. Protective Effect of Glutamine Peptides on Chemical Barrier

The chemical barrier is mainly composed of mucus secreted by intestinal epithelial cells, digestive fluid, and bacteriostatic substances secreted by commensal bacteria. Intestinal mucosal epithelium is interspersed with a large number of goblet cells, which can secrete a large amount of mucus and form a translucent mucus layer. It is also the first physical defense line that external molecules encounter after entering the intestine [77]. The main component of the mucus layer is mucin, which plays a crucial role in protecting and maintaining the integrity of the epithelial physical barrier in the colon [78]. Currently, nearly twenty kinds of mucin have been identified, such as Mucin2, Mucin6, and Mucin19. A recent study has shown that the damage to intestinal barrier function caused by mycotoxins was correlated to the decrease in mucin secretion [79]. Glutamine peptides can protect the chemical barrier by regulating the number of goblet cells and the level of mucin [69]. Both glutamine and alanyl-glutamine can reduce the down-regulation effect of zearalenone on MUC-2 mRNA expression, thereby improving intestinal epithelial barrier dysfunction [80]. Similar results were obtained in the turbot intestinal disease model caused by soybean meal, and the expression of MUC-2 and ppar-γ genes was significantly increased by Ala-Gln [75]. Similarly, a study by Hou demonstrated that prophylactic use of alanyl-glutamine dipeptide can significantly up-regulate the expression of mucin-2, trefoil factor-3, and heat shock protein-72, effectively promote the repair of DSS-induced mucosal injury, and protect the chemical barrier [81]. In conclusion, effective regulation of mucin expression is an effective means to combat intestinal chemical barrier damage.

The mucus layer exists in the form of a single layer and double layer in the small intestine and colon, respectively, and is generally distributed on the surface of intestinal mucosa [77]. The outer layer is loose and rich in bacteria and bacterial products, while the inner layer is sterile and firmly attached to the surface of the epithelial cells [82]. The mucus layer separates the epithelial cells from the host tissues and symbiotic bacteria and can effectively prevent bacteria and other harmful substances from entering the surface of intestinal epithelium [83]. In addition, mucopolysaccharide, lysozyme, and glycoprotein are also contained in the mucopolysaccharide layer, which together restrict the proliferation and colonization of bacteria.

### 4.3. Protective Effect of Glutamine Peptides on Immunological Barrier

The immunological barrier is primarily constituted by the gut-associated lymphoid tissue (GALT) and diffusely distributed immune lymphocytes. GALT includes intraepithelial lymphocytes, lamina propria lymphocytes, and intestinal lymph nodes. These lymphoid tissues are mainly distributed among intestinal mucosal epithelial cells, lamina propria, and submucosa. When exogenous toxic substances invade the intestine and act as antigens to trigger an immune response in the intestinal mucosa, the immune barrier becomes activated. It recognizes these foreign antigens through the intestinal mucosal immune system and employs substances secreted by lymphoid tissues and cells to selectively eliminate the antigens, thereby fulfilling its immune barrier function to safeguard intestinal health [84,85]. Lymphocytes mainly include T lymphocytes, B lymphocytes, macrophages, dendritic cells, and natural killer cells. They defend against and monitor antigens by secreting regulatory cytokines and immunoglobulins.

Glutamine peptides can be used as the energy supplier of cells, and effectively promote the mitosis, differentiation, and proliferation of lymphocytes and macrophages. The proliferation and activation of immune cells can effectively regulate the synthesis and secretion of inflammatory cytokines, thus regulating immune homeostasis. In addition, it is well known that excess production of pro-inflammatory factors can exacerbate the inflammatory cascade, causing intestinal damage and damaging the immune barrier [86]. Increasing evidence shows that glutamine peptide plays a role in protecting the immune barrier by regulating cytokine levels. Anti-inflammatory peptides obtained from germinated soybean protein are rich in a variety of glutamine peptides, such as QQQQQGGSQSQ, QEPQESQQ, QQQQQGGSQSQKG, PETMQQQQQQ, which can effectively inhibit cellular inflammatory response and enhance immune barrier function [59]. Our previous research confirmed that corn protein hydrolysate with glutamine-rich peptides effectively regulates cytokine levels, including reducing the pro-inflammatory cytokines IL-1β, IL-6, and TNF-α and increasing the anti-inflammatory cytokine IL-10, to improve DSS-induced colitis in mice [57]. NF-κB is one of the markers of inflammatory response and has been associated with many diseases, such as immune response and inflammatory response [87]. NF-κB-signaling pathway is also one of the main signaling pathways mediating inflammatory response. It is mediated by Toll-like receptor 4 (TLR4), and induces intracellular signal transduction through myeloid differentiation factor 88 (MyD88), activates NF-κB, and leads to increased secretion of inflammatory cytokines [88,89]. A recent study showed that dietary alanyl-glutamine supplementation can effectively down-regulate the expression of myd88, NF-κB and pro-inflammatory factor genes in hybrid grouper, inhibit the NF-κB signaling pathway, and enhance the immune barrier to defend against colitis [90]. The polypeptide (DMPIQAFLLYQEPVLGPVR) identified from bovine β-casein has the activity of inhibiting NF-κB and has a good antagonistic effect on inflammatory reaction induced by TNF-α [91]. Therefore, inhibition of the NF-κB-signaling pathway may be an important target for glutamine peptides to enhance the immune barrier.

On the other hand, oxidative stress is closely related to inflammation. Oxygen free radicals are the inducers of inflammatory response, which can accelerate the release of inflammatory factors and aggravate the damage of inflammatory response [92]. It is worth noting that it has been proven that oxidative stress-induced apoptosis of intestinal cells is the inducement of up-regulation of intestinal permeability [93]. Glutamine peptides can play a role in protecting the immune barrier from the perspective of reducing oxidative stress. IQW, a glutamine peptide derived from egg white protein ovotransferrin, has good anti-inflammatory and antioxidant activity and can alleviate TNF-α-induced endothelial cell inflammatory response and oxidative stress [63]. In addition, IQW has also been shown to enhance the intestinal immunological barrier by reducing TNF-α and IL-17 levels in a mouse model of DSS-induced colitis, which is a novel option for the prevention of inflammatory bowel disease [94]. Gu et al. investigated the protective effect of alanyl-glutamine on intestinal barrier dysfunction through an in vitro cell model. The study revealed that alanyl-glutamine dipeptide can attenuate zearalenone-induced cytotoxicity, apoptosis, and increased para-cellular permeability, while also alleviating zearalenone-induced barrier function impairment by improving the antioxidant capacity of cells, including reducing ROS production and increasing GSH levels [80]. Glutamine peptide (AVPYPQ) identified from β-casein could effectively reduce ROS levels in IEC-6 cells treated with hydrogen peroxide [95]. These studies indicated that glutamine peptides can play a role in protecting the immunological barrier by regulating levels of inflammatory cytokines and reducing oxidative stress.

### 4.4. Protective Effect of Glutamine Peptides on the Biological Barrier

The biological barrier is primarily composed of two components: the luminal microbiota and the mucosal microbiota. The luminal microbiota is dominated by bacteria, such as *Escherichia coli* and *Enterococcus*, while the mucosal microbiota mainly includes *Bifidobacterium* and *Lactobacillus*. These microbial communities adhere to the intestinal mucosal layer, forming a multi-layered intestinal microbial barrier [96]. Intestinal flora and the intestinal environment have formed a relatively stable symbiotic relationship after long-term evolution. On the one hand, the gut can provide nutrition and a reproductive environment for the intestinal flora; on the other hand, intestinal flora can reduce intestinal permeability, increase the epithelial defense mechanism, assist carbohydrate fermentation, and form a biological barrier to protect the intestinal tract [97]. In addition, the number and distribution of microorganisms in the intestinal flora are also relatively constant, and the microbial flora is relatively balanced and stable. Under normal circumstances, the resident bacteria in the intestinal tract will not cause diseases, but when stimulated by external factors, such as immunity and stress, the number and activity of the intestinal flora may change, the flora will shift, and the proportion of beneficial bacteria and harmful bacteria will be unbalanced, thus causing the destruction of the biological barrier [98,99]. The changes in intestinal flora are involved in the formation of obesity, non-alcoholic fatty liver disease, inflammatory bowel disease, and many other diseases [100,101,102].

Multiple studies have shown that glutamine peptide can alleviate the damage of biological barriers by regulating the community diversity, evenness, richness, and composition of intestinal flora. For example, Alanyl-glutamine dipeptide alleviates DSS-induced colitis in mice by regulating intestinal flora, including decreasing the ratio of Bacteroidetes/Firmicutes and enhancing the functions of the microflora metabolism pathways [60]. Similar results were also obtained by a previous study that glycyl-glutamine alleviated intestinal barrier damage caused by the weaning of piglets by regulating intestinal flora [62]. Corn-protein-fermented feed rich in glutamine peptide can significantly improve the intestinal health level of broilers and increase the number of beneficial bacteria, especially Lactobacillus, thus playing a role in protecting intestinal biological barrier function [103]. In addition, glutamine peptides can significantly increase the content of beneficial bacteria during the regulation of intestinal flora, while the major metabolites of beneficial bacteria are short-chain fatty acids (SCFAs). It has been reported that the role of SCFs in protecting intestinal barrier function is mainly achieved by reducing the pH in the intestinal environment to inhibit the proliferation of pathogens and increase the expression of intestinal epithelial cells’ tight junction protein [104]. Therefore, this appears to be another mechanism by which glutamine peptides protect the biological barrier.

In summary, glutamine peptides have demonstrated promising effects in regulating the aforementioned four barriers. However, current research, as reported, has primarily focused on in vivo animal models and in vitro cell models, often with small sample sizes and a notable lack of clinical human trials. It is well-recognized that inherent differences exist between clinical trials and animal models, including variations in dosage and bioavailability of glutamine peptides. Therefore, more large-scale clinical studies are warranted to further validate the actual efficacy of glutamine peptides in humans.

## 5. Concluding Remarks and Future Perspectives

Glutamine peptide, a bioactive peptide, is widely present in humans, animals, plants, and microorganisms. It plays a crucial role in modulating intestinal barrier function and maintaining intestinal health. These biological effects are primarily mediated through the regulation of tight junction proteins, mucin secretion, inflammatory responses, and gut microbiota composition. Despite the significant progress made in the study of glutamine peptides, many issues still need to be addressed. Firstly, more attention needs to be paid to the preparation of natural glutamine peptides, including improving the extraction rate, as well as the separation and purification of glutamine peptides. At present, most researchers still use synthetic glutamine dipeptides as the research object to study their physiological function in the human body or animal body, while as mentioned above, the synthetic method has the disadvantages of high cost, complicated process, environmental pollution and so on. Only a few scholars have prepared glutamine peptides by enzymatic hydrolysis of plant protein, but the extraction rate and purity are low. Therefore, future studies in this research field need to be emphasized. Moreover, there are still some challenges regarding the molecular regulatory mechanisms of glutamine peptide protection of intestinal barrier function. Currently, the commonly used models to evaluate the function of glutamine peptide to protect the intestinal barrier include the in vitro Caco-2 cell model and in vivo colitis model. However, these models can only reflect whether glutamine peptides have a protective effect on barrier function, and the specific mechanism of action has not been fully elucidated. At present, studies on the signaling pathway mainly focus on some recognized pathways, such as MLCK/MLC and TLR4/MyD88/NF-κB, while other signaling pathways and mechanisms are unclear and need to be further explored. In addition, the structure–activity relationships of glutamine peptides and their transport and absorption mechanism in the gut are still limited. As we all know, bioactive peptides need to be completely absorbed by the intestine or digested into active fragments by the gastrointestinal tract and then enter the blood circulation to reach the target organ, so as to play a role in the body. Therefore, studying the structure–activity relationship and bioavailability of glutamine peptides is of great significance for further understanding their role in protecting intestinal barrier function.

## Figures and Tables

**Table 1:** Commonly used food-grade protease.

Protease	Pepsin	Trypsin	Papain	Flavourzyme	Alcalase	Neutrase	Protamex
Property	Endopeptidase	Endopeptidase	Endopeptidase	Endopeptidase/Exopeptidase	Endopeptidase	Endopeptidase	Endopeptidase
Action site	Leu-Phe-	Lys-Arg-	Asn-Gln, Glu-Ala, Leu-Val	—	Ala-Leu-	—	—
Temperature	37	37	45–75	50	55–70	45–55	35–60
pH	1.8–3.0	6.0–9.0	5.0–7.0	5.0–7.0	6.5–8.5	5.5–7.5	5.5–7.5

**Table 2:** Common methods for preparation of glutamine peptides.

Method	Advantage	Limitation
Chemical synthesis	High efficiency, high product purity, and excellent stability	High cost, complex operation, and pollute the environment
Enzymatic hydrolysis	Simple operation and mild reaction conditions	Low efficiency, non-unique product, and low purity

**Table 3:** Common methods for determination of glutamine in glutamine peptides.

Method	Advantage	Limitation
Enzymatic Hydrolysis	Simple operation and mild reaction conditions	An indirect method for determination and only suitable for determination of short peptides
Edman Degradation	A direct determination method with accurate results	High cost, complex operation, and only suitable for single peptide samples
BTI Derivative	Simple operation and no special restrictions on the samples	An indirect method for determination and only suitable for determination of non-*N*-terminal glutamine

**Table 4:** Glutamine peptides or protein hydrolysates with glutamine-rich peptides with activities to regulate intestinal barrier function.

Peptides/Hydrolysates	Sequence	Model/Method	Main Mechanism	References
Collagen peptide	GPSGPQGSR	In vitro model of TNF-α treated Caco-2 cell monolayers	Increased TEER of Caco-2 cell monolayer and decreased its permeability	(Song et al., 2019) [56]
Corn protein hydrolysate with glutamine-rich peptides	A total of 374 corn glutamine peptides	A mouse model of DSS-induced colitis; In vitro model of LPS-treated Caco-2 cell	Reduced the permeability of the colonic mucosa in mice, regulated the abundance and diversity of the intestinal microbiota; Up-regulated the gene expression of tight junction proteins, and regulated levels of inflammatory cytokines	(Jing et al., 2022; Jing et al., 2024) [57,58]
Peptides derived from in vitro gastrointestinal digestion of germinated soybean proteins	QQQQQGGSQSQ, QEPQESQQ, QQQQQGGSQSQSQKG, PETMQQQQQQ	In vitro model of LPS-treated RAW267.4	Reduced inflammatory response	(Marcela et al., 2018) [59]
Peptide	AQ	A mouse model of DSS-induced colitis	Decreased levels of inflammatory cytokines and increased the expression of occludin; Modulated gut microbiota and microflora metabolites	(Xu et al., 2021; Liu et al., 2023) [60,61]
Peptide	GQ	A Piglet model	Modulated the gut microbiota and microflora metabolites	(Yan et al., 2020) [62]
Peptide derived from egg white protein ovotransferrin	IQW	In vitro model of TNF-induced endothelial cells	Inhibited the up-regulation of intercellular cell adhesion molecule-I	(Majumder et al. 2013) [63]
Peptide derived from pepsin–soy protein hydrolysates	EKPQQQSSRRGS	In vitro model of LPS-treated RAW267.4	Enhance immune regulation	(Hsieh et al. 2022) [64]
Peptide	CQ, VQ	A mouse model of DSS-induced colitis; In vitro model of TNF-α treated Caco-2 cell	Inhibit the expression of inflammatory cytokines	(Zhang et al. 2015) [65]
Peptide derived from Crassostrea gigas	QCQCAVEGGL	A mouse model of DSS-induced colitis	Decreased serum IgE levels and increased spleen CD4^+^/ CD8^+^ levels	(Hwang et al. 2012) [66]

## Data Availability

All data generated for this research are included in the article.
